# School leadership and indigenous student outcomes: A correlational study of Student Needs Management Practices based on Maslow’s Hierarchy of Needs

**DOI:** 10.1371/journal.pone.0350479

**Published:** 2026-07-30

**Authors:** Yusoff Yahaya, Bity Salwana Alias, Mohd Norazmi Nordin

**Affiliations:** 1 Faculty of Education, Universiti Kebangsaan Malaysia, Bangi, Selangor, Malaysia; 2 Centre for Educational Leadership and Policy Research, Universiti Kebangsaan Malaysia, Bangi, Selangor, Malaysia; 3 Centre for Community Education and Wellbeing Research, Universiti Kebangsaan Malaysia, Bangi, Selangor, Malaysia; Akademia Jagiellońska w Toruniu: Akademia Jagiellonska, CZECHIA

## Abstract

Indigenous students continue to face persistent educational disadvantages, including lower academic attainment and reduced school engagement, often linked to school systems that do not systematically address students’ hierarchical needs. This study examined the association between principal-led Student Needs Management Practices (SNMP) and Indigenous student outcomes across 93 national primary schools in Orang Asli settlement areas in Peninsular Malaysia. Using a quantitative cross-sectional design, data were collected from 342 teachers through multi-stage sampling. SNMP was operationalised across five Maslow-derived dimensions physiological, safety, belongingness, esteem, and self-actualisation needs while Indigenous student outcomes encompassed academic achievement, co-curricular participation, and character development. Both SNMP (M = 4.63, SD = 0.39) and student outcomes (M = 4.26, SD = 0.49) were rated at very high levels by teacher respondents. Pearson correlation revealed a significant, moderate positive association between SNMP and student outcomes (r = .411, p < .001). Multiple regression indicated that the five SNMP dimensions collectively explained 18.8% of the variance in student outcomes (R^2^ = .188, adjusted R^2^ = .176), F(5, 336) = 15.559, p < .001. Belongingness needs emerged as the only statistically significant predictor (β = .298, p < .001); safety needs approached significance (β = .124, p = .061); physiological, esteem, and self-actualisation needs were not significant predictors. These findings suggest that emotional and relational dimensions of leadership practice are more directly associated with Indigenous student outcomes than material or recognition-based dimensions, and that the modest direct association is consistent with leadership-influence models in which principals affect student outcomes substantially through intermediate mechanisms such as teacher commitment. The study offers evidence-based implications for needs-responsive school leadership in Indigenous education contexts.

## Introduction

The educational trajectories of Indigenous students remain a persistent challenge for policymakers, practitioners, and researchers worldwide. International bodies such as UNESCO and the OECD consistently document that Indigenous learners experience pronounced disadvantages in educational participation, attainment, and resource access relative to non-Indigenous peers [1]. Although Sustainable Development Goal 4 (SDG 4) commits to inclusive and equitable quality education, progress in addressing Indigenous educational disparities remains uneven [2,3].

In Malaysia, Orang Asli students in settlement-area national primary schools face compounded structural disadvantages, including geographic isolation, socio-economic constraint, and cultural dissonance between home and school environments [4–6]. School leadership is theorised to play a pivotal role in mitigating these disadvantages, yet most leadership research in Indigenous contexts has examined macro-level policy and access factors rather than how individual school leaders operationalise responsiveness to students’ hierarchical needs at the school level [7].

The concept of Student Needs Management Practices (SNMP) defined here as the systematic, theoretically grounded efforts of school principals to identify and address students’ physiological, safety, belongingness, esteem, and self-actualisation needs has received limited empirical attention as a structured leadership construct, despite its conceptual grounding in Maslow’s Hierarchy of Needs [8]. Existing research linking leadership practices to Indigenous student outcomes has tended to rely on single-dimension outcome measures or qualitative description, leaving the relative contribution of distinct need dimensions to multidimensional student outcomes (academic, co-curricular, and character-related) empirically underexamined [9].

This study addresses this gap by examining the association between principal-led SNMP and Indigenous student outcomes using a quantitative, cross-sectional design and multiple regression analysis. It is important to note at the outset that, consistent with the cross-sectional nature of the design, the analyses reported here establish statistical association and relative predictive contribution rather than causal influence; no causal claims are made or implied. The study contributes empirical evidence on which dimensions of need-responsive leadership are most strongly associated with Indigenous student outcomes, situated within the broader literature on indirect models of leadership influence [10].

## Literature review

### Theoretical foundation: Maslow’s Hierarchy of Needs and its critiques

Maslow’s Hierarchy of Needs [11] remains a widely applied framework for understanding human motivation, positing a progression from physiological needs through safety, belongingness, esteem, and ultimately self-actualisation, with lower-order need fulfilment theorised as a prerequisite for higher-order functioning. In educational contexts, the framework offers a lens for understanding how unmet foundational needs may constrain engagement and achievement, particularly among learners facing socio-economic adversity [12].

The framework is not without limitations, and contemporary scholarship has questioned several of its core assumptions. The strict hierarchical and sequential ordering of needs has limited cross-cultural empirical support; need fulfilment across categories often occurs concurrently rather than sequentially, and the relative salience of specific needs varies substantially across cultural and community contexts [13]. Critics have also noted that Maslow’s model was developed without reference to collectivist or non-Western value systems, raising questions about its direct applicability to Indigenous communities in which relational and communal needs may take precedence over individual self-actualisation in ways the original model does not anticipate [14]. This concern is consistent with broader critiques of the epistemological mismatch between Western-derived theoretical frameworks and Indigenous standpoints; Nakata’s [15] concept of the cultural interface highlights the tensions that arise when Indigenous educational experience is interpreted solely through theoretical lenses developed outside, and without reference to, Indigenous knowledge systems. The present study treats Maslow’s hierarchy as a heuristic for organising dimensions of need-responsive leadership practice rather than as a strictly sequential causal model, and interprets its findings, where the data depart from the theorised hierarchy, as an empirical contribution to this critical discourse rather than a disconfirmation to be explained away.

### Student Needs Management Practices in educational leadership

SNMP refers to the systematic efforts of school leaders to identify, address, and sustain students’ physical, emotional, social, and developmental needs within the school environment. The construct aligns with student-centred and transformational leadership orientations that position holistic learner development as a central leadership function [16], and resonates closely with the culturally responsive school leadership (CRSL) framework, which similarly calls for school environments not merely classroom instruction to be made responsive to the schooling needs of minoritised and marginalised students through critical self-awareness, community advocacy, and inclusive school climates [17]. Principals are theorised to influence student outcomes both directly, through the school conditions they create, and indirectly, through their effect on teacher capacity, motivation, and commitment a distinction formalised in Leithwood, Harris and Hopkins’ [10] synthesis of indirect leadership influence, which finds that school leaders’ effects on student outcomes are substantially mediated through teacher-level mechanisms rather than exerted directly. Recent evidence reinforces this mediated pathway, showing that distributed school leadership enhances teacher job satisfaction primarily through its effect on teacher collaboration rather than through a direct relationship [18], underscoring the relevance of teacher-level mechanisms to the SNMP–outcome pathway examined in the present study.

### Indigenous education and holistic student development

Indigenous student success is increasingly conceptualised beyond narrow academic attainment to encompass social competence, cultural identity, and character development [19,20]. Bronfenbrenner’s ecological systems theory [21] further suggests that Indigenous student outcomes are shaped by multiple nested systems from the microsystem of family and peers to the macrosystem of policy and community culture many of which lie outside the direct influence of any single school leader; evidence that family nurturing environments and early home conditions shape children’s developmental trajectories well before school entry similarly underscores the limits of any single school-level leadership practice in fully accounting for student outcomes [22]. This perspective implies that a single leadership-practice variable should be expected to explain only a modest, rather than a dominant, share of variance in student outcomes, and that a modest but statistically significant association still represents a meaningful empirical finding within this complex causal landscape.

Beyond the Indigenous-specific literature, broader evidence on inclusive education systems reinforces the centrality of belonging-oriented conditions for student success: a realist evaluation of a territorial inclusive-education innovation found that when systemic conditions enabled students to experience safety, full class membership, and motivation to learn, learning progress and social participation improved markedly, even for learners with complex developmental needs [23]. This finding anticipates the present study’s emphasis on belongingness as the dimension most strongly associated with Indigenous student outcomes.

Comparative international scholarship from countries with established Indigenous education research traditions offers substantial precedent for situating the present study. In Australia, a systematic review synthesised evidence on how school and community leadership contributes to Aboriginal student learning and social outcomes, identifying collaborative decision-making and sustained engagement as central mechanisms [24]; principals in remote and rural Indigenous-serving schools have similarly been positioned as ‘protagonists’ whose practices of cultural validation, holistic wellbeing, and reciprocal community relationships shape Indigenous student participation and achievement [25], while recent theorising has extended this leadership work toward ‘culturally nourishing’ models that move beyond Western bureaucratic structures [26]. This body of work is further contextualised by scholarship on the legacy of racism and identity within Indigenous Australian education, cautioning against interpreting Indigenous educational disadvantage through purely Western epistemological frames [27].

In Aotearoa New Zealand, principal-level culturally responsive leadership has been shown to support Māori student achievement in urban primary schools [28], and a large-scale mixed-methods study across 84 secondary schools identified persistent impediments to implementing culturally responsive leadership, including limited partnership with Indigenous students and their communities [29]. More recent quantitative analysis of leadership practices across high-performing secondary schools has directly linked culturally responsive leadership behaviours to positive University Entrance attainment rates among Māori and Pacific students [30], while capacity-building models emphasising cross-cultural, Indigenous-led collaboration have been proposed to strengthen school leaders’ culturally responsive practice [31].

In Canada, recent scholarship has called for the infusion of Métis and First Nations worldviews, particularly the relational principle of Wâhkôhtowin, into K–12 education leadership frameworks as a basis for decolonising school practice [32], complementing multi-case evidence that principals’ emphasis on student belonging, relationship-building, and culturally relevant school experiences is seminal to positive outcomes for Aboriginal learners [33], broader work on Indigenous student success through culturally responsive practices in Ontario [34], school-board-level case evidence that investment in students’ socio-emotional wellbeing and cultural identity, while necessary, may be insufficient without sustained attention to academic achievement [35], and Indigenous knowledge-informed school community relationality in rural Alberta [36]. Despite this substantial and growing body of work, comparatively little empirical attention particularly using quantitative, multivariate methods at the primary and secondary school level has been directed toward principal-level leadership practices addressing the hierarchical needs of Indigenous learners in Southeast Asian contexts [37] such as Malaysia, a gap the present study addresses.

### SNMP, teacher commitment, and student outcomes

A growing body of evidence links teacher–student relationships and teacher commitment to student outcomes; Hattie’s synthesis of influences on achievement [38] places constructs closely related to affective and normative teacher commitment among the most influential correlates of student achievement. This raises the possibility that principal-led SNMP may be associated with student outcomes both directly and indirectly via teacher commitment, consistent with indirect-effects models of school leadership [10]. The present study focuses on the direct association between SNMP and student outcomes; the indirect pathway through teacher commitment, while theoretically relevant and supported by preliminary descriptive evidence within the broader research programme, is not formally tested here and is identified as a priority for future mediation analysis.

### Conceptual framework and hypotheses

**Fig 1:**
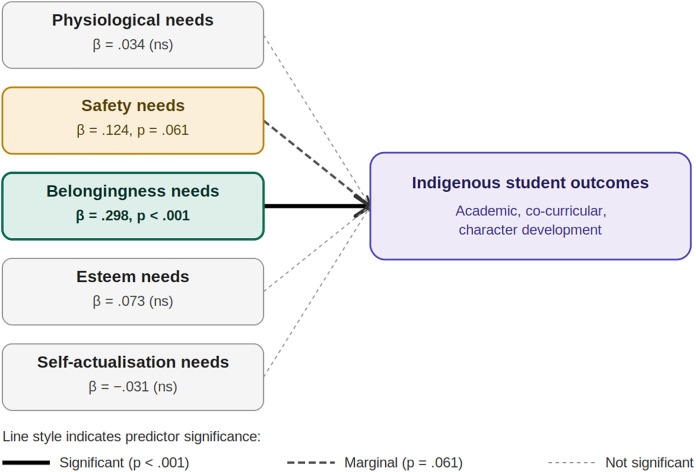
Conceptual framework of Student Needs Management Practices (SNMP) and Indigenous student outcomes. Five SNMP dimensions are positioned as predictor variables of the composite Indigenous student outcomes variable. Line style indicates the strength of statistical association observed in the regression results reported in Table 1: belongingness needs (solid line) was the only statistically significant predictor; safety needs (dashed line) approached significance; physiological, esteem, and self-actualisation needs (thin dotted lines) were not statistically significant. The framework is presented here for descriptive and interpretive purposes and does not imply a confirmatory structural equation model.

The conceptual framework positions SNMP operationalised across five dimensions (physiological, safety, belongingness, esteem, self-actualisation needs) as the set of predictor variables, and Indigenous student outcomes (academic achievement, co-curricular participation, character development) as the composite outcome variable (Fig 1). Based on the literature reviewed, the following hypotheses were tested:

**Table 1:** Multiple regression analysis predicting Indigenous student outcomes from SNMP dimensions (N = 342).

Predictor variable	B	Beta (β)	t	p	Tolerance	VIF
(Constant)	1.924	–	6.366	< .001	–	–
Physiological needs	0.027	.034	0.578	.564	0.695	1.438
Safety needs	0.141	.124	1.879	.061	0.557	1.796
Belongingness needsᵃ	0.289	.298	3.623	< .001	0.357	2.798
Esteem needs	0.083	.073	0.951	.342	0.407	2.459
Self-actualisation needs	−0.034	−.031	−0.347	.729	0.306	3.272

R = .434, R^2^ = .188, adjusted R^2^ = .176; F (5, 336) = 15.559, p < .001.

ᵃStatistically significant predictor (p < .05).

B = unstandardised coefficient; Beta = standardised coefficient; VIF = Variance Inflation Factor.

*H1: There is a significant positive association between overall SNMP and Indigenous student outcomes.*

*H2a–H2e: Physiological (H2a), safety (H2b), belongingness (H2c), esteem (H2d), and self-actualisation (H2e) need each make a significant contribution to predicting Indigenous student outcomes.*

## Materials and methods

### Research design and ethical approval

A quantitative, cross-sectional survey design with a correlational analytical framework was employed. Ethical approval for this research was obtained from the Educational Planning and Research Division (Bahagian Perancangan dan Penyelidikan Dasar Pendidikan), Ministry of Education Malaysia (approval reference: KPM.600-3/2/3-eras (22854)), covering data collection across seven states. As the research was conducted entirely within Ministry of Education schools and involved Ministry personnel (teachers) as respondents, Ministry-level ethical approval constituted the primary and required institutional clearance for this study; a separate university-level Institutional Review Board (IRB) approval was not separately sought, as it was not a parallel requirement for this category of ministry-based educational research at the time of data collection. Participation was voluntary, anonymous, and based on written informed consent, with respondents informed of their right to withdraw at any time.

### Population and sampling

The study population comprised teachers across 93 national primary schools located in Orang Asli settlement areas across three zones of Peninsular Malaysia (Tengah/Perak, Timur/Pahang, and Selatan/Johor). A sample of 342 teachers was obtained through multi-stage sampling combining cluster, stratified random, and simple random sampling, with sample size determined according to the Krejcie and Morgan [39] sampling table, supplemented by an additional buffer to account for anticipated non-response. This approach was intended to ensure adequate representation across the three geographical zones and participating schools, supporting the generalisability of the findings within this population.

### Instrumentation

SNMP was measured using an adapted questionnaire comprising 33 items across five dimensions: physiological needs (5 items, e.g., “Guru Besar di sekolah saya menyediakan kemudahan makanan percuma kepada murid Orang Asli yang memerlukan” [“The principal at my school provides free meal facilities for Orang Asli students in need”]), safety needs (6 items, e.g., “Guru Besar di sekolah saya sentiasa mengingatkan murid Orang Asli agar melaporkan jika terdapat sebarang gangguan atau ancaman terhadap keselamatan” [“The principal at my school consistently reminds Orang Asli students to report any safety threats or disturbances”]), belongingness needs (6 items), esteem needs (8 items), and self-actualisation needs (8 items). Indigenous student outcomes were measured using 17 items across academic achievement (8 items, referenced to classroom assessment [PBD] and end-of-year examination [UASA] performance in core subjects), co-curricular participation (4 items), and character development (5 items). All items used a five-point Likert scale (1 = Strongly Disagree to 5 = Strongly Agree) and were completed by class or subject teachers reporting on their observations of students in their care.

Instrument validity was established through a three-stage process. Face validity was assessed by three Malay-language experts with over 10 years’ experience, who reviewed item clarity and phrasing. Content validity was assessed via the Content Validity Index (CVI) using a three-member expert panel (a university lecturer in educational management, a Ministry of Education officer experienced in Orang Asli education, and an Institut Aminuddin Baki lecturer specialising in school leadership), following Polit and Beck’s [40] procedure and Lynn’s [41] minimum acceptance threshold of 0.78 per item. Average CVI across the three experts was 0.92 (Expert 1 = 0.92, Expert 2 = 0.85, Expert 3 = 0.98), exceeding the minimum threshold. Based on expert feedback, four items were refined for clarity of language without altering the underlying construct structure.

Construct validity was further assessed via Rasch Measurement Model analysis (Winsteps version 5.10.3) in a pilot study (n = 130). Item infit mean-square was 0.95 and outfit mean-square was 1.00, both within Linacre’s [42] accepted range of 0.5–1.5, indicating adequate item fit. Person reliability was 0.90 and item reliability ranged from 0.75 to 0.77. The point-measure correlation between raw scores and Rasch logit measures was 0.96, providing further evidence of measurement structure coherence.

Internal consistency reliability (pilot study, n = 130) was assessed using Cronbach’s alpha for each sub-construct: physiological needs α = .788, safety needs α = .977, belongingness needs α = .918, esteem needs α = .990, self-actualisation needs α = .989, academic achievement α = .981, co-curricular participation α = .961, and character development α = .922. Corrected item-total correlations ranged from .455 to .986 across all constructs, exceeding the conventional .30 minimum threshold, and no items required removal.

### Common method considerations

Because both SNMP and student outcome measures were reported by the same respondents (teachers) using the same instrument and response format, the design carries an inherent risk of common method bias (CMB). Several procedural remedies recommended by Podsakoff et al. [43] were incorporated at the design stage: respondent anonymity was assured to reduce social desirability and evaluation apprehension; predictor and outcome items were presented in separate, clearly delineated sections of the questionnaire rather than interleaved; and item wording was reviewed by the expert panel to minimise ambiguity and shared semantic priming between constructs. A statistical CMB diagnostic was subsequently conducted using Harman’s single-factor test, extracting one component from all 63 items (33 SNMP items and 17 student outcome items and 13 teacher commitment items included in the broader survey). The single factor accounted for 41.86% of the total variance (Eigenvalue = 26.372), which is below the conventional 50% threshold for serious CMB concern, providing supplementary evidence that common method variance is unlikely to account for the observed associations. Nonetheless, the procedural-only nature of CMB mitigation and the absence of multi-source outcome data remain acknowledged limitations. The student outcome measure reflects teachers’ perceptions and classroom-based assessment records as reported by teachers, rather than independently verified student records; all results should be interpreted within this scope.

The sample design involved 342 teachers nested within 93 schools, which raises the question of whether the independence assumption of ordinary multiple regression was met. To assess the potential clustering effect, the design effect (DEFF) was estimated using the formula DEFF = 1 + (ṅ − 1) × ICC, where ṅ denotes the mean cluster size and ICC denotes the intraclass correlation coefficient. The dataset contains a school code identifier (Kod Sekolah) for 34 of the 93 participating schools (the remaining schools were recorded under grouped codes). Using these 34 identifiable school clusters (k = 34, N = 342, ṅ = 10.06), a one-way ANOVA was conducted on the composite student outcome score. The between-group mean square was MS_B = 0.238 and the within-group mean square was MS_W = 0.222, yielding F (33, 308) = 1.069, p = .371. The empirical ICC was calculated as ICC = (MS_B − MS_W) ÷ (MS_B + (ṅ − 1) × MS_W) = .007, indicating that only 0.7% of the variance in student outcomes is attributable to between-school differences. The corresponding DEFF = 1 + (10.06 − 1) × .007 = 1.063, which falls well below the conventional threshold of 2.0 above which clustering is considered a substantive threat to standard error estimates [44], confirming that the independence assumption of ordinary multiple regression is not materially violated in this sample and that OLS regression remains the appropriate analytical approach.

### Data analysis

Data were analysed using SPSS version 30.0. Descriptive statistics characterised the level of SNMP and student outcomes. Pearson correlation examined the bivariate association between SNMP and student outcomes. Multiple regression assessed the relative contribution of the five SNMP dimensions to student outcomes. Prior to regression, five assumptions were tested. Normality was assessed via skewness and kurtosis for each variable (N = 342): values for most variables fell within the ± 1.96 acceptable range [45], although safety needs (kurtosis = −0.581), physiological needs (kurtosis = 2.281), belongingness needs (kurtosis = 3.004), and academic achievement (kurtosis = 3.246) showed mild leptokurtic departures; given the large sample size (N > 200), such minor deviations were not considered consequential for parametric analysis [46]. Regression residuals were further assessed: Kolmogorov–Smirnov and Shapiro–Wilk tests were statistically significant (p < .001), which is common with large samples and highly sensitive to trivial deviations; residual skewness (−0.160) and kurtosis (2.575) remained within an acceptable practical range, and visual inspection of the histogram and normal P-P plot showed an approximately normal, symmetric distribution. Linearity and homoscedasticity were assessed via scatterplots of standardised predicted values against standardised residuals, which showed a random scatter without curvilinear or funnel-shaped patterning, supporting both assumptions. Independence of residuals was supported by a Durbin–Watson statistic of 1.626, within the conventional acceptable range of 1.5–2.5. Multicollinearity was assessed via Tolerance and Variance Inflation Factor (VIF): Tolerance exceeded 0.1 and VIF ranged from 1.438 to 3.272 across predictors, well below the conventional threshold of 5, indicating no serious multicollinearity by this criterion. As a more conservative cross-check, eigenvalue-based collinearity diagnostics were also examined: the condition index for the smallest eigenvalue dimension reached 54.76, exceeding Belsley, Kuh and Welsch’s [47] conventional concern threshold of 30, with elevated variance proportions concentrated in self-actualisation needs (.87) and belongingness needs (.49) on this dimension. We report this transparently as a more sensitive, and partially discordant, collinearity signal than VIF/Tolerance alone suggested; it indicates some shared variance between these two predictors at higher dimensions, though coefficient signs and significance patterns remained stable and theoretically interpretable, and is noted as a limitation warranting caution in interpreting the relative independence of these two specific coefficients. Case-wise diagnostics indicated no unduly influential observations: Cook’s Distance ranged from .000 to .363 (well below the conventional concern threshold of 1.0), and centred leverage values ranged from .002 to .147.

Multiple regression, rather than structural equation modelling (SEM) or partial least squares SEM (PLS-SEM), was adopted as the primary analytical approach because the study’s objective was to estimate the relative predictive contribution of five conceptually distinct, individually validated Maslow-derived dimensions to a composite outcome variable, rather than to estimate a latent measurement model with multiple interrelated constructs and structural paths. We acknowledge that SEM or PLS-SEM would offer advantages where SNMP and Indigenous student outcomes are treated as latent variables measured with error and where mediating pathways (e.g., through teacher commitment) are modelled explicitly; this is identified as a priority direction for future research building on the present findings, particularly given the indirect-effects literature discussed above [10].

## Results

### Descriptive analysis

Overall SNMP was rated at a very high level (M = 4.63, SD = 0.39). Among the five dimensions, esteem needs recorded the highest mean (M = 4.71, SD = 0.43), followed closely by safety needs (M = 4.70, SD = 0.43), self-actualisation needs (M = 4.67, SD = 0.45), belongingness needs (M = 4.56, SD = 0.51), and physiological needs (M = 4.43, SD = 0.63), which recorded the lowest, though still very high, mean. Overall Indigenous student outcomes were also rated at a very high level (M = 4.26, SD = 0.49); co-curricular participation recorded the highest sub-dimension mean (M = 4.47, SD = 0.49), followed by character development (M = 4.34, SD = 0.57) and academic achievement (M = 4.09, SD = 0.67), the latter being comparatively lower and showing the greatest score dispersion.

### Correlation analysis

Pearson correlation revealed a statistically significant, moderate, positive association between overall SNMP and Indigenous student outcomes (r = .411, p < .001, N = 342), supporting H1. Following Jackson’s [48] interpretive convention, in which coefficients between +.30 and +.69 indicate a moderate relationship, this association is interpreted as moderate in strength. The zero-order correlation matrix among the five SNMP predictor dimensions is presented in Table 2.

**Table 2:** Zero-order Pearson correlations among the five SNMP dimensions (N = 342).

Dimension	1	2	3	4	5
1. Physiological needs	–	.389**	.532**	.447**	.463**
2. Safety needs	.389**	–	.602**	.588**	.601**
3. Belongingness needs	.532**	.602**	–	.639**	.759**
4. Esteem needs	.447**	.588**	.639**	–	.743**
5. Self-actualisation needs	.463**	.601**	.759**	.743**	–

** p < .001 (two-tailed).

Inter-predictor correlations range from .389 to .759. The highest correlations are between belongingness needs and self-actualisation needs (r = .759) and between esteem needs and self-actualisation needs (r = .743), consistent with the eigenvalue-based collinearity diagnostics reported in the Methods section.

### Multiple regression analysis

Multiple regression was conducted to assess the relative contribution of the five SNMP dimensions to Indigenous student outcomes. The overall model was statistically significant,F (5, 336) = 15.559, p < .001, accounting for 18.8% of the variance in student outcomes (R = .434, R^2^ = .188, adjusted R^2^ = .176). Full coefficients, including multicollinearity diagnostics, are reported in Table 1.

Among the five SNMP dimensions, only belongingness needs made a statistically significant contribution to predicting Indigenous student outcomes (β = .298, p < .001), supporting H2c. Safety needs approached but did not reach conventional significance (β = .124, p = .061), providing only marginal support for H2b. Physiological needs (β = .034, p = .564; H2a not supported), esteem needs (β = .073, p = .342; H2d not supported), and self-actualisation needs (β = −.031, p = .729; H2e not supported) were not significant predictors. Tolerance and VIF values for all predictors fell within acceptable limits, confirming that the non-significance of four predictors reflects their statistical relationship with the outcome rather than multicollinearity artefacts. In terms of overall effect size, the model corresponds to Cohen’s f^2^ = .232, falling within the medium-to-large range [46]. With the zero-order correlation matrix now available (Table 2), the squared semi-partial correlation (sr^2^) for belongingness needs the sole significant predictor was estimated as sr^2^ ≈ .037 (i.e., approximately 3.7% unique variance contribution), calculated from the t-statistic as sr^2^ = (t^2^ × (1 − R^2^)) / (N − k − 1), or equivalently as (β × r_y1) − (sum of other products), indicating that belongingness needs accounts for approximately 3.7% of the total variance in Indigenous student outcomes uniquely, above and beyond the shared variance with other predictors. The high inter-predictor correlations between belongingness needs and self-actualisation needs (r = .759) and between esteem needs and self-actualisation needs (r = .743) explain the suppression of these predictors’ individual betas, consistent with the collinearity diagnostic pattern noted above.

Overall, while the full SNMP construct shows a significant moderate association with Indigenous student outcomes (H1 supported), the regression results indicate that this association is driven primarily by the belongingness dimension, with safety needs showing a secondary, marginal contribution.

## Discussion

This study examined the association between principal-led SNMP and Indigenous student outcomes. Consistent with H1, a significant moderate positive correlation was found between overall SNMP and student outcomes (r = .411, p < .001). However, regression analysis revealed that this aggregate association is attributable almost entirely to a single dimension: belongingness needs.

The primacy of belongingness as the sole significant predictor of Indigenous student outcomes is a substantively important finding that runs counter to a strict reading of Maslow’s sequential hierarchy, in which lower-order needs (physiological, safety) would be expected to dominate prediction of outcomes in a disadvantaged population. Instead, the data suggest that emotional connection, social inclusion, and relational warmth the practices captured by the belongingness dimension are more directly associated with Indigenous students’ academic, co-curricular, and character outcomes than material provision, physical safety, recognition, or self-actualisation practices. This pattern is consistent with critiques of Maslow’s framework noted earlier, which argue that relational and communal needs may take precedence in collectivist and Indigenous cultural contexts rather than following a strictly sequential, individualistic ordering [13,14]. It also resonates with Hattie’s [38] identification of teacher–student relationship quality as among the most influential correlates of student achievement, and with evidence that the social composition of the classroom and the degree of friendship segregation among students shape the broader peer-belonging environment within which leadership practices operate [49], suggesting that the relational mechanisms underlying both constructs may be closely related.

Safety needs approached significance (p = .061) and may warrant cautious interpretation as a secondary contributor; however, as this did not reach the conventional .05 threshold, no firm conclusion is drawn, and replication with a larger or different sample would be needed to determine whether this represents a true, if modest, effect or sampling variability.

The non-significance of physiological, esteem, and self-actualisation needs as direct predictors does not imply these dimensions are unimportant to Indigenous student welfare; rather, it suggests their association with the specific outcome measures used here (academic, co-curricular, and character indicators, as reported by teachers) may be indirect, mediated through other mechanisms such as belongingness itself, teacher commitment, or longer-term developmental pathways not captured in a cross-sectional design. Evidence that targeted nutritional interventions can meaningfully enhance school children’s micronutrient intake and, by extension, their capacity to learn [50] suggests that physiological provisioning remains practically important even where it does not emerge as a statistically significant direct predictor of the composite outcome measure used here. This interpretation aligns with Bronfenbrenner’s ecological systems theory [21], which frames Indigenous student outcomes as shaped by multiple nested systems beyond any single school-level leadership practice; the modest R^2^ (.188) is therefore consistent with theoretical expectations rather than indicative of measurement failure.

The moderate (rather than strong) direct association between SNMP and student outcomes (r = .411) is also consistent with indirect models of leadership influence [10], under which principals’ effects on student outcomes operate substantially through intermediate mechanisms such as teacher commitment and capacity, rather than through a strong direct pathway. Descriptive correlational evidence from the broader research programme indicates a stronger association between SNMP and teacher commitment (r = .749) and between teacher commitment and student outcomes (r = .432) than between SNMP and student outcomes directly, a pattern that is suggestive of, but does not formally establish, a mediated pathway; this possibility was not the focus of the present analysis and is identified as a priority for future research employing formal mediation modelling (e.g., via structural equation modelling).

Several limitations should be acknowledged. First, common method bias remains a plausible concern despite the Harman single-factor diagnostic result (41.86% variance explained by one factor, below the 50% threshold) and the procedural remedies applied; future research should employ multi-source outcome data (e.g., independent student records) to reduce reliance on teacher-reported measures for both predictor and outcome constructs. Second, the student outcome measure reflects teachers’ perceptions and classroom-based assessment records as reported by teachers themselves, rather than independently verified student data; all results should be interpreted within this scope. Third, the cross-sectional design precludes causal inference; all associations reported here should be interpreted as correlational. Fourth, school identifiers were not recorded as a variable in the dataset, precluding a formal multilevel model; the empirical ICC computed from school cluster data available in the dataset was .007, yielding DEFF = 1.063, confirming that clustering effects did not materially bias the regression standard errors in this sample. A formal multilevel model with complete school-level identifiers for all 93 schools is nevertheless recommended in future replication studies. Fifth, while multiple regression was appropriate for the present objective, structural equation modelling or PLS-SEM would offer a more comprehensive test of the underlying measurement and mediation structure, including the potential mediating role of teacher commitment, in future research.

## Conclusion

This study found a significant moderate association between principal-led SNMP and Indigenous student outcomes, with belongingness needs emerging as the only dimension significantly associated with outcomes in regression analysis. These findings suggest that relational and emotional dimensions of school leadership practice warrant particular attention in efforts to support Indigenous student development, without discounting the theoretical and practical importance of safety, physiological, esteem, and self-actualisation needs, whose contribution may operate through indirect or longer-term pathways not captured in this cross-sectional analysis.

Practically, these findings suggest that school leaders in Indigenous education settings may benefit from prioritising relational practices that foster students’ sense of belonging and emotional connection to the school community, alongside continued attention to safety, material support, and recognition as part of a holistic leadership approach. At the policy level, professional development for school leaders in Indigenous and other marginalised-community contexts could usefully incorporate relational and socio-emotional leadership competencies alongside administrative and welfare-oriented training.

This study is limited by its cross-sectional design, its reliance on teacher-reported data for both predictor and outcome constructs, and the resulting risk of common method bias. Future research employing longitudinal designs, multi-source or independently verified student outcome data, and formal mediation analysis involving teacher commitment would substantially strengthen causal and mechanistic understanding in this area.

## Supporting information

S1 File PLOS questionnaire on inclusivity in global research. Completed questionnaire on inclusivity in global research for this study involving Orang Asli Indigenous populations in Peninsular Malaysia.
(DOCX)
