# Supplementary material for: School leadership and indigenous student outcomes: A correlational study of Student Needs Management Practices based on Maslow’s Hierarchy of Needs
Source: PLoS One. 2026 Jul 30;21(7):e0350479. doi: 10.1371/journal.pone.0350479 (PMC13422855; doi:10.1371/journal.pone.0350479)
Supplement: S1 File — Completed questionnaire on inclusivity in global research for this study involving Orang Asli Indigenous populations in Peninsular Malaysia. (DOCX) [file pone.0350479.s001.docx]

**PLOS Questionnaire on Inclusivity in Global Research**

*Completed for manuscript PONE-D-26-22994*

*"School Leadership and Indigenous Student Outcomes: A Correlational Study of Student Needs Management Practices Based on Maslow’s Hierarchy of Needs"*

*Additional information regarding the ethical, cultural, and scientific considerations specific to inclusivity in global research is included in this Supporting Information file, as referenced in the “Inclusivity in global research” subsection of the Methods section of the main manuscript.*

**Section 1: Ethical Considerations, Permits, and Authorship**

(Applicable to all research types)

**1.1 Provide details as to who granted permissions and/or consent for the study to take place. This should include the names of all ethics boards, governmental organizations, community leaders, or other bodies that provided approval for the study (refer to roles/titles, not personal names).**

*Response:*

Ethical approval for this study was granted by the Educational Planning and Research Division (Bahagian Perancangan dan Penyelidikan Dasar Pendidikan, BPPDP), Ministry of Education Malaysia (approval reference: KPM.600-3/2/3-eras(28194)), covering data collection across seven states of Peninsular Malaysia. Institutional access and on-site permission for each of the 93 participating national primary schools were additionally granted by the respective school principals (Guru Besar) as the designated institutional gatekeepers. No separate university-level Institutional Review Board approval was sought, as Ministry-level approval constituted the required and primary institutional clearance for research conducted within Ministry of Education schools involving Ministry personnel as respondents.

**1.2 If there were any deviations from the study protocol after approval was obtained, please provide details of these changes.**

*Response:*

Not applicable. No deviations from the approved study protocol occurred after ethical approval was obtained.

**1.3 Did this study involve local collaborators that are residents of the country where the research was conducted or members of the community studied? If you do not have any authors from said communities, please provide an explanation for this.**

*Response:*

Yes. All three authors (Yusoff Yahaya, Bity Salwana Alias, and Mohd Norazmi Nordin) are Malaysian nationals affiliated with Universiti Kebangsaan Malaysia (UKM), and the corresponding author and first author hold professional roles within the Malaysian Ministry of Education with direct, ongoing professional engagement in Orang Asli education. This is therefore a wholly domestic research project conducted by local researchers studying communities within their own country, rather than research involving travel by foreign researchers to study an external community or population. The research team did not, however, include co-authors who are themselves members of the Orang Asli community; this is acknowledged as a limitation, and is addressed further in Section 2 below.

**Section 2: Human Subjects Research**

(e.g. health research, medical research, cross-cultural psychology, educational and social science research)

**2.1 Did you obtain written informed consent from a representative of the local community or region before the research took place? How did you establish who speaks for the community?**

*Response:*

The direct human participants in this study were 342 teachers serving in national primary schools located in Orang Asli settlement areas; Orang Asli community members, students, or families were not themselves directly recruited as study respondents, as the student outcome construct was assessed via teacher report rather than direct engagement with Orang Asli students or their guardians. Accordingly, formal consent processes were structured around the institutional and professional context of the respondents: written informed consent was obtained individually from each teacher participant, and institutional access was authorised by the Ministry of Education Malaysia and individual school principals as the recognised governing authorities for school-based research. A separate, distinct community-level consent process involving Orang Asli community leaders or representatives (e.g., Tok Batin) was not undertaken, as the research did not directly involve Orang Asli individuals as data sources. We acknowledge this as a limitation of the present design and recommend that future research extending this work to directly involve Orang Asli students, families, or community representatives as participants or co-designers should establish community-level consent through recognised Orang Asli community leadership structures.

**2.2 How did members of the local community provide input on the aims of the research investigation, its methodology, and its anticipated outcome(s)?**

*Response:*

The research aims and methodology were developed by the research team based on review of the academic literature on Indigenous education, school leadership, and Maslow’s Hierarchy of Needs, and were refined through consultation with a three-member expert panel comprising a university lecturer in educational management, a Ministry of Education officer experienced specifically in Orang Asli education, and an Institut Aminuddin Baki lecturer specialising in school leadership. The Ministry of Education officer’s involvement provided a degree of policy-level and practitioner familiarity with the Orang Asli education context during instrument development. However, direct input from Orang Asli community members or representatives on the research aims, methodology, or anticipated outcomes was not formally sought. We acknowledge this as a limitation and recommend that future research in this area incorporate structured community consultation, for example through engagement with Jabatan Kemajuan Orang Asli (JAKOA) or recognised community representatives, at the research design stage.

**2.3 When engaging with the local community, how did you ensure that the informed consent documents and other materials could be understood by local stakeholders?**

*Response:*

As the direct study respondents were professional teachers rather than Orang Asli community members directly, informed consent materials were prepared in Bahasa Malaysia, the shared professional working language of all respondents. Both the consent materials and the survey instrument itself underwent face validity review by three Malay-language experts with more than ten years’ professional experience, who assessed clarity of language, sentence structure, and terminological accuracy to ensure comprehensibility for the teacher respondent population.

**2.4 Will the findings of the research be made available in an understandable format to stakeholders in the community where the study was conducted (e.g. via a presentation, summary report, copies of publications, etc.)? Please provide details of how this will be achieved.**

*Response:*

Yes. The authors intend to prepare a Bahasa Malaysia-language summary report of the key findings for distribution to the participating schools and the relevant divisions of the Ministry of Education Malaysia (including the Jemaah Nazir dan Jaminan Kualiti and the Educational Planning and Research Division) following publication. The corresponding author additionally intends to share findings through professional networks engaged in Orang Asli education policy and practice, to support practical uptake of the study’s implications for school leadership in Indigenous education settings.

**Section 3: Non-Human Subjects Research**

(e.g. research using specimens/animals collected as part of the study, or those housed in archival collections; examples include archaeology, paleontology, botany, and zoology)

**3.1–3.4**

*Response:*

Not applicable. This study did not involve the collection, use, or analysis of biological specimens, animals, archaeological materials, or archival collections. All data were collected via teacher questionnaire and principal interview within an educational research design.
